# A survey on the use of continuous positive airway pressure in newborn care in Kenya in 2017–2018

**DOI:** 10.1371/journal.pone.0322310

**Published:** 2025-04-30

**Authors:** Jemma L. Wright, Emma Haddon, Helen M. Nabwera, Fiona M. Dickinson, Mary-Jo Hoare, Pamela Godia, Judith Maua, Mercy K. Sammy, Bridget C. Naimoi, Onesmus Muchemi, Sylvia Kawira, Joyce Mutuku, Osman H. Warfa, Beatrice Ochieng, Sophie Ngugi, Allan Govoga, Florence Murila, Alexander Manu, William M. Macharia, Matthews Mathai, Juan E. Dewez

**Affiliations:** 1 Flintshire Children’s Centre, Betsi Cadwaladr University Health Board, Mancot, United Kingdom; 2 City St George's, University of London, London, United Kingdom; 3 Liverpool School of Tropical Medicine, Pembroke Place, Liverpool, United Kingdom; 4 Centre of Excellence for Women and Child Health and Department of Paediatrics, Aga Khan University, Nairobi, Kenya; 5 Liverpool School of Tropical Medicine, Nairobi, Kenya; 6 Department of Public and Global Health, University of Nairobi, Nairobi, Kenya; 7 Gertrude’s Children’s Hospital, Nairobi, Kenya; 8 Department of Paediatrics and Child Health, University of Nairobi, Nairobi, Kenya; 9 Ministry of Health, Division of Newborn and Child Health, Nairobi, Kenya; 10 London School of Hygiene and Tropical Medicine, London, United Kingdom,; University of Cape Town Department of Paediatrics and Child Health, SOUTH AFRICA

## Abstract

**Background:**

Globally, complications of preterm birth are the leading cause of under-5-mortality. Respiratory distress syndrome (RDS) is a common and life-threatening complication among preterm infants. Continuous positive airway pressure (CPAP) is a relatively simple and effective intervention that is recommended for RDS treatment. However, appropriate infrastructure and processes are required to ensure that it is used safely, effectively and sustainably. This study describes how CPAP was used in newborn care in Kenya between 2017–2018. Our aim was to identify enablers, barriers and gaps in CPAP use.

**Methods:**

A cross-sectional survey was carried out across all newborn baby units in Kenya between 2017–2018, as part of a evaluation of CPAP use in newborn care. Descriptive statistics were used to analyse the quantitative data.

**Results:**

Twenty-three hospitals across 15 (32%) of the counties in Kenya were providing CPAP in newborn care. The survey was conducted in 19 hospitals, amounting to 83% of all hospitals providing CPAP in newborn care in the country. Sub-county (level 4) and county (level 5) referral had fewer resources (i.e., trained staff, infrastructure and equipment) than the national referral (level 6) and private hospitals. In addition, there was a wide variation in the CPAP devices used and the resources for supporting CPAP use across different hospitals.

**Conclusion:**

We found access to CPAP for neonates with RDS was inequitable in Kenya. There were also disparities in the availability of resources, personnel, and guidelines to support its implementation. Lack of standardisation of CPAP use in newborn care was especially evident in the public sector. To optimise coverage and standardisation of CPAP use in newborn care in Kenya, our results support ongoing partnerships to strengthen public and private healthcare sectors involving the implementation of strategies to improve infrastructure for newborn care, train and retain staff, and provide additional equipment.

## Introduction

In 2022, there were 2.3 million newborn deaths, and the majority were due to preventable or treatable causes [1]. It is known that the neonatal period (< 28 days of life) is the most vulnerable period of a child’s life [2] and three quarters of newborn deaths occur within the first seven days of life [3, 4]. Although the mortality rate in the under 5’s has more than halved in recent years, the reduction in neonatal deaths has been slower [5]. As a result, neonatal causes now account for nearly half of under 5 deaths globally [6]. As nearly 80% of neonatal deaths occur in sub-Saharan Africa and southern Asia [6], a greater focus on addressing the challenges faced in optimising newborn health in these regions is vital.

The three leading causes of neonatal deaths are preterm birth associated complications, intrapartum-related asphyxia or trauma, and neonatal sepsis [1, 3, 4]. Preterm birth accounts for 35% of newborn deaths [6, 7] and an estimated 15 million babies are born preterm each year [8]. Respiratory distress syndrome (RDS) is the dominant clinical condition facing preterm newborns and is invariably life-threatening, in the absence of evidence-based life-saving interventions [8–10].

Continuous positive airway pressure (CPAP) is a non-invasive method of providing respiratory support to newborns and has been used to treat RDS in high income settings since 1971 [9]. There are many different CPAP delivery devices available with varying complexity and cost [9, 10]. Typical CPAP delivery methods in low- and middle-income countries (LMICs) include mechanical ventilators with a CPAP mode, bubble CPAP, flow driven CPAP and home-made variations [10–13].

CPAP is recommended as a key intervention for use in LMICs to end preventable newborn deaths by 2030 [13, 14]. In 2014, a pivotal non-randomised study from Malawi compared the impact of a low-cost bubble CPAP system with oxygen therapy (the standard of care) in preterm and low birth weight newborns with severe respiratory distress. It reported an absolute improvement in survival of 27% for newborns that received bubble CPAP [15]. Subsequently, other implementation studies in Africa have reported improved newborn survival with CPAP use, even in rural district hospitals [16–18].

Despite the potential life-saving role of CPAP in treating RDS in preterm infants in LMICs, it is important to consider the potential adverse effects that can be associated with CPAP use that may be missed or overlooked in the context of limited resources. These include the risk of pneumothorax, nasal trauma, and necrotising enterocolitis [10, 19, 20]. In the long-term, unregulated oxygen use in newborns can result in complications including retinopathy of prematurity (ROP) and bronchopulmonary dysplasia (BPD) [20–22], which are difficult to diagnose and treat in LMICs [20]. A randomised controlled trial of bubble CPAP use in children with severe pneumonia, malnutrition and HIV in Malawi was stopped prematurely by the Data Safety and Monitoring Committee as the results revealed that bubble CPAP was associated with increased risk of mortality [23]. This study emphasised that CPAP outcomes depend on proper patient selection, supportive infrastructure and implementation by skilled healthcare professionals in low resource settings. These findings were corroborated in a systematic review by Kinshella et al that sought to evaluate the barriers and facilitators of CPAP implementation for newborn care in sub-Saharan African health facilities in addition to the staffing shortages and sub-optimal engagement of caregivers during treatment [24].

To address the high neonatal mortality rate of 21 per 1000 live births in Kenya [25] and accelerate progress towards achieving the sustainable development goal of reducing neonatal mortality rate to below 12 per 1000 live births by 2030 [26], the Ministry of Health in Kenya has been working to scale up CPAP use in newborn care [27, 28]. Despite the improvement in newborn service delivery resulting from the Newborn Essential Solutions and Technologies (NEST360, https://nest360.org/), an international alliance that seeks to implement packages of care to end preventable newborn deaths in African hospitals, there has been limited change in most newborn care units across Kenya since our period of data collection [29, 30].

The aim of this study was to describe the use of CPAP in newborn care across Kenya, including the availability of infrastructure and processes for CPAP administration in healthcare facilities. Our intention in 2017/2018 was to identify the enablers, barriers and gaps to CPAP use in order to inform future scale up of CPAP as part of Every Newborn Action Plan (ENAP). Our study preceded the NEST360 project.

This paper complements the previously published qualitative data from this evaluation, which provides more in-depth insights into health care worker experiences of using CPAP in newborn care in this setting [31].

## Methods

### Study design

This was a cross-sectional survey using standard questionnaires across newborn baby units (NBUs) in Kenya. The recruitment period for this study was between 1^st^ September 2017 and 28^th^ February 2018. These questionnaires were designed using the WHO standards for improving the quality of newborn care in health facilities [32].

### Study setting

The study was conducted across newborn baby units providing inpatient secondary or tertiary level newborn care services in which CPAP was used. The government-funded public healthcare system in Kenya involves two tiers; the county government oversees primary and secondary services at county level (including county referral hospitals and regional referral hospitals), and the national government oversees the national referral hospitals [33] Government funded public healthcare accounts for 48% of healthcare in Kenya with the remaining 41% accounted for by private for profit (private) and 11% by private not for profit (faith) providers [34]. At the time of the study, it was estimated that each of the 47 Counties of Kenya had at least one NBU, at the county referral and/or regional referral hospital. Less than half (18/47, 38%) of NBUs in public hospitals in Kenya provided CPAP (see S1 Fig)

### Study population and sampling

**Fig 1:**
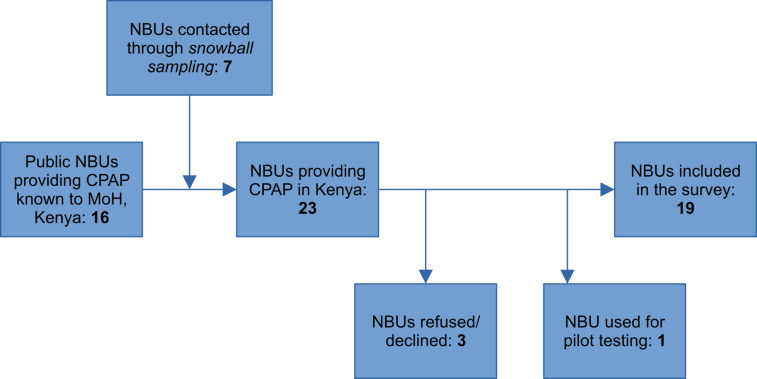
Sampling process.

Identification of the hospitals was based on information provided by the Division of Child and Adolescent Health at the Ministry of Health of Kenya and non-governmental organisations (NGOs) involved in newborn care in Kenya. Sixteen public hospitals were identified through the Ministry of Health and a further seven hospitals were identified through a process of snowball sampling, where senior clinicians provided details of other hospitals using CPAP in newborn care [35]. Twenty-three hospitals were identified as providing CPAP in newborn care (18 public, 3 private and 2 mission). Three hospitals (2 public and 1 private) declined to take part in the evaluation, and 1 was used to pilot the survey tool. Therefore, the survey was conducted across the 19 hospitals with CPAP in newborn care, which were spread across 15 (32%) of the 47 counties of Kenya, see Fig 1.

For each participating hospital, senior members of medical and nursing staff were selected to participate due to their clinical experience and knowledge of infrastructure and processes within their respective facilities. This method of “expert sampling” (a type of purposive sampling) involves selecting individuals who are experts within the subject area to provide further insight and illuminate new topics of interest [36]. Although this method is more commonly used in qualitative research, it was used in the study due to the exploratory nature of the evaluation.

### Data collection

A standard survey questionnaire (‘Newborn Baby Unit CPAP Assessment Tool’) was used in each newborn unit. This questionnaire was developed by researchers at the Centre for Maternal and Newborn Health, Liverpool School of Tropical Medicine in collaboration with newborn health experts in India and the UK based on international standards for CPAP use in newborn care. Further details about the creation of this questionnaire, following the Donabedian framework for assessing quality of healthcare, have already been published [12]. In summary, the questionnaire covered nine domains of assessment based on WHO standards for CPAP use in newborn care [32]. It included 136 questions pertaining to availability of structure (infrastructure), processes (practice of care) and outcomes (clinical outcomes) related to CPAP use. The questionnaire was piloted in one newborn unit in Kenya and revised before commencement of data collection.

The field team were five research assistants with a medical background and experience in maternal and child health. They received training on the WHO Essential Newborn Care strategies [37] and the role and safe use of CPAP in newborn care by the principal investigator. The members of the team with a nursing background observed the use of CPAP in the Queen Elizabeth Hospital in Blantyre, Malawi to gain experience in the use of a low-cost bubble CPAP device in newborn care. The whole team also received training on communication skills in research and health, good clinical practice and data collection.

The research assistants administered the questionnaire in person with a senior member of the medical or nursing team at each newborn care unit between September 2017 and February 2018. Data was captured electronically using password protected electronic tablets using FileMaker Pro software (FileMaker Inc.).

### Data management and statistical analysis

Data were extracted from FileMaker Pro into an Excel spreadsheet. Any data queries were addressed with the field team before descriptive statistical analyses were performed using IBM SPSS Statistics 26. Summary statistics including frequencies, means/standard deviations for normally distributed data, and median/interquartile ranges for non-normally distributed data were used to describe the data.

### Ethics approval

The study was approved by the research and ethics committees of the Liverpool School of Tropical Medicine (15–032) and the Kenyatta National Hospital/ University of Nairobi (P56/02/2017). Further permission was obtained at each participating health facility following authorisation by the national Ministry of Health. A full explanation of the study was given to each participant highlighting the voluntary nature of participation and that specific information from each facility would be treated confidentially and anonymised after collection. Each participant was provided with a participation information sheet before obtaining consent. Informed written consent was obtained from all participants and recorded on consent forms prior to participation in the study. No minors were involved in the study.

## Results

### Distribution of CPAP in newborn care in Kenya

The distribution of CPAP in NBUs varied depending on the county with a range of 1–6 NBUs per county. At the time of our evaluation, the most populous county (Nairobi County) had six hospitals that provided CPAP in NBUs, whilst most of the other counties only had one hospital with this intervention.

### CPAP devices used in newborn care in Kenya

**Fig 2:**
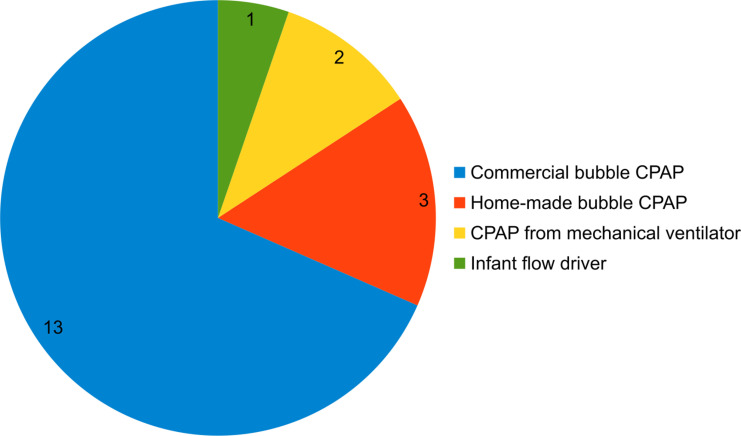
CPAP devices used in newborn care.

We found that commercial CPAP devices were the most common mode of delivering CPAP in newborn care in Kenya (17/19, 89%). These systems included commercial devices, i.e., devices manufactured by industry including bubble CPAP (13/19, 68%), infant flow driver systems (1/19, 5%), and mechanical ventilators with a CPAP mode (2/19, 11%). The two remaining faith-based hospitals used indigenous (home-made) CPAP systems (2/19, 11%) (Fig 2).

### Duration of CPAP use in newborn care in Kenya

Overall, the NBUs had used CPAP for a median of 3 years (IQR 2–5 years). Private hospitals (median 9 years, IQR 6–12 years) and faith-based hospitals (median 11.5 years, IQR 7–16 years) had used CPAP for longer period than public hospitals. One private hospital had used CPAP for 16 years.

### Infrastructure for CPAP use in Kenya

#### Physical infrastructure.

Reliable emergency electrical power was available in most NBUs apart from in the four public county referral hospitals. Technical maintenance of biomedical equipment was not available in one public county referral hospital and three regional referral hospitals, equating to 21% of the NBUs surveyed. Both air or oxygen were available in 79% (15/19) of NBUs, whilst oxygen only was available in faith-based hospitals (2/19, 11%) and an external gas supply (i.e., oxygen provided through a pipe from a central source or gas cylinder) required for certain CPAP devices was only available in 68% (13/19) of the NBUs (Table 1).

**Table 1:** Physical infrastructure available, per hospital type.

Infrastructure	National Referral Hospital n (%)	Regional Referral Hospital n (%)	County Referral Hospital n (%)	Private Hospital n (%)	Total n (%)
Reliable emergency electrical power	1 (100)	6 (100)	4 (50)	4 (100)	15 (79)
Technical maintenance of biomedical equipment	1 (100)	3 (50)	7 (88)	4 (100)	15 (79)
Air or oxygen available	1 (100)	6 (100)	6 (75)	2 (50)	15 (79)
External gas supply available	1 (100)	4 (67)	6 (75)	2 (50)	13 (68)
CPAP-specific breathing circuits	1 (100)	6 (100)	6 (75)	2 (50)	15 (79)
CPAP-specific nasal prongs	1 (100)	6 (100)	8 (100)	2 (50)	17 (89)
CPAP system equipped with oxygen-air blender	1 (100)	4 (67)	3 (38)	2 (50)	10 (53)
CPAP system equipped with humidifier	1 (100)	2 (33)	3 (38)	4 (100)	10 (53)

CPAP-specific infrastructure tended to be present in private hospitals and public national referral hospitals but was lacking in the faith-based hospitals. The availability varied in the public regional and country hospitals (Table 1). The number of NBUs with access to CPAP specific breathing circuits was 15/19 (79%) and CPAP-specific nasal prongs was 17/19 (89%). Only 53% (10/19) of NBUs had CPAP systems equipped with oxygen-air blenders or humidifiers.

#### Staffing numbers and skill-base.

All NBUs had at least one doctor available 24 hours a day but only 84% had at least one doctor competent in newborn resuscitation available 24 hours a day. This percentage further reduced to 32% of NBUs that had at least one doctor trained in CPAP available 24 hours a day. Similarly, 89% NBUs had at least one nurse competent in newborn resuscitation available 24 hours a day and only 32% NBUs had at least one nurse trained in CPAP available 24 hours a day. Fewer than half of NBUs (8, 42%) provided CPAP training for staff. Faith-based hospitals had no CPAP training at all, meaning that only one private hospital had CPAP training available, which was provided as part of an allocated workplace induction. Seven of the public hospitals (47%) had CPAP training available that was provided as ‘on the job’ clinical training. Overall, the median number of nurses per NBU shift was 2 (IQR 1–3). Each nurse was expected to care for a median of 31 infants (IQR 11–50) per shift including a median of 2 infants (IQR 1–2) on CPAP (Table 2).

**Table 2:** Staffing available, per hospital type.

Infrastructure	National Referral Hospital n (%)	Regional Referral Hospital n (%)	County Referral Hospital n (%)	Private Hospital n (%)	Total n (%)
At least one doctor available 24hrs/day	1 (100)	6 (100)	8 (100)	4 (100)	19 (100)
At least one doctor competent in newborn resuscitation available 24hrs/day	1 (100)	6 (100)	5 (63)	4 (100)	16 (84)
At least one CPAP-trained doctor available 24hrs/day	1 (100)	2 (33)	2 (25)	1 (25)	6 (32)
At least one nurse competent in newborn resuscitation available 24rs/day	1 (100)	6 (100)	6 (75)	4 (100)	17 (89)
At least one CPAP-trained nurse on each shift	1 (100)	2 (33)	2 (25)	1 (25)	6 (32)

#### Access to essential medicines.

Essential medicines related to CPAP use include caffeine, surfactant, intravenous fluids and antibiotics. All NBUs had access to intravenous fluids (10% Dextrose/0.9% Normal Saline) as well as common first line antibiotics (Benzylpenicillin/Gentamycin). Caffeine and surfactant were available in 21% and 26% of NBUs respectively. These two medications were available in the national referral hospital but not in the other public hospitals. Private hospitals had access to surfactant, but the faith-based hospitals did not have access to caffeine. Antenatal steroids were available in 89% of the NBUs (Table 3).

**Table 3:** Essential medication available, per hospital type.

Infrastructure	National Referral Hospital n (%)	Regional Referral Hospital n (%)	County Referral Hospital n (%)	Private Hospital n (%)	Total n (%)
Availability of caffeine	1 (100)	1 (17)	0 (0)	2 (50)	4 (21)
Availability of surfactant	1 (100)	0 (0)	0 (0)	4 (100)	5 (26)
Availability of antenatal corticosteroids	1 (100)	5 (83)	8 (100)	3 (75)	17 (89)
Availability of cryotherapy or laser therapy for retinopathy of prematurity	1 (100)	0 (0)	0 (0)	3 (75)	4 (21)

#### Support services and equipment for CPAP.

Key monitoring support services for CPAP use include radiology, haemoglobin testing, and blood culture analysis. These services were available in private hospitals but were limited in public hospitals. Overall, 58% of NBUs had 24 hour access to radiology, 100% had 24 hour access to haemoglobin testing, 100% had access to blood sugar monitoring and 63% had access to blood culture analysis. Access to important bedside monitoring, such as pulse oximeters, was low (79%) (Table 4).

**Table 4:** Support services and essential equipment available, per hospital type.

Infrastructure	National Referral Hospital n (%)	Regional Referral Hospital n (%)	County Referral Hospital n (%)	Private Hospital n (%)	Total n (%)
Availability of pulse oximeters	1 (100)	4 (65)	6 (75)	4 (100)	15 (79)
X-ray available 24hr/day	1 (100)	3 (50)	3 (38)	4 (100)	11 (58)
Haemoglobin testing available 24hr/day	1 (100)	6 (100)	8 (100)	4 (100)	19 (100)
Blood culture analysis available	1 (100)	5 (83)	2 (25)	4 (100)	12 (63)
Availability of functioning bag and masks in immediate care area	1 (100)	6 (100)	7 (88)	4 (100)	18 (95)
Availability of mechanical ventilation	1 (100)	3 (50)	0 (0)	3 (75)	7 (37)
Availability of transilluminator	0 (0)	1 (17)	0 (0)	0 (0)	1 (5)
Availability of neonatal-specific intercostal catheter insertion for management of pneumothorax	1 (100)	1 (17)	0 (0)	3 (75)	5 (26)

The availability of functioning emergency equipment in the immediate care area for the resuscitation of newborns via bag and mask was present in 95% of NBUs. However mechanical ventilation, which is the typical escalation of care for newborns requiring prolonged bag and mask resuscitation or struggling on CPAP, was only available in 37% of NBUs. All hospitals expect the public national hospital had systems in place to refer neonatal patients to hospitals delivering a higher level of care. This was because the public national hospitals were the highest form of care available in Kenya (Table 4).

A transilluminator was available in one public regional hospital, equating to 5% of NBUs. Similarly, only 26% of NBUs had neonatal-specific intercostal catheters available for the management of pneumothorax. This meant that most of the NBUs did not have the equipment to diagnose or treat pneumothoraxes in newborns, which is a common complication of CPAP. Only 21% of NBUs had access to cryotherapy or laser therapy for the treatment of ROP (Table 4).

### Processes for CPAP use in newborn care in Kenya

#### Availability of newborn care guidelines for CPAP.

**Fig 3:**
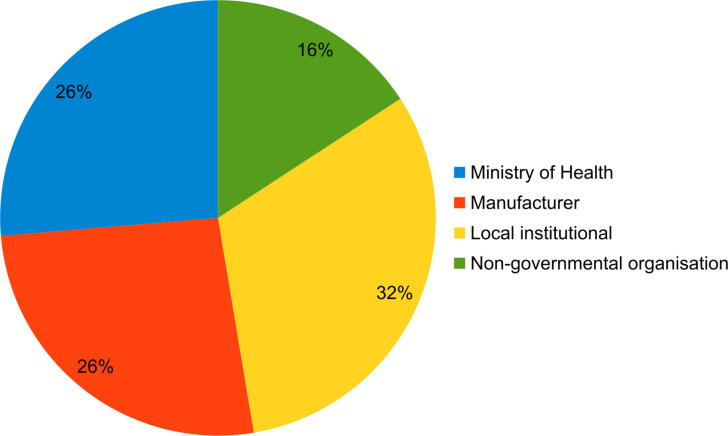
Source of guidelines for using CPAP in newborn care units.

The majority of NBUs (84%) had CPAP guidelines available from sources such as the Ministry of Health and equipment manufacturers (Fig 3). Only public hospitals used government guidelines from the Ministry of Health, which was comprised of a one-page protocol [27]. The private hospitals all used international guidelines.

#### Initiation of CPAP in newborns.

All NBUs had criteria for initiating CPAP, including indications and contraindications for using CPAP, but these varied between hospital types (Table 5). Respiratory distress was an indication for CPAP use in all NBUs, however there was variation in the other listed indications. There was no agreement regarding the contraindications for CPAP use (Table 6). Furthermore, there was no standardisation between hospitals in the criteria for starting CPAP.

**Table 6:** Contraindications for starting CPAP in newborns, per hospital type.

Contraindication	National Referral Hospital n (%)	Regional Referral Hospital n (%)	County Referral Hospital n (%)	Private Hospital n (%)	Total n (%)
Pneumothorax	0 (0)	1 (17)	0 (0)	3 (75)	4 (21)
Unstable respiratory drive	1 (100)	5 (83)	3 (38)	2 (50)	11 (58)
Tracheosophageal fistula	0 (0)	4 (67)	2 (25)	3 (75)	9 (47)
Gastroschisis	0 (0)	3 (50)	2 (25)	2 (50)	7 (37)
Necrotising Enterocolitis	0 (0)	1 (17)	0 (0)	2 (50)	3 (16)
Cleft Palate	0 (0)	5 (83)	4 (50)	2 (50)‍	11 (58)
Comatose/Hypotonia	0 (0)	2 (33)	4 (50)	0 (0)	6 (32)

**Table 5:** Indications for starting CPAP in newborns, per hospital type.

Indication	National Referral Hospital n (%)	Regional Referral Hospital n (%)	County Referral Hospital n (%)	Private Hospital n (%)	Total n (%)
Respiratory Distress	1 (100)	67 (100)	8 (100)	4 (100)	19 (100)
Recurrent Apnoea	1 (100)	3 (50)	2 (25)	3 (75)	9 (47)
Support post-extubation	0 (0)	1 (17)	0 (0)	3 (75)	4 (21)
Pneumonia	0 (0)	2 (33)	4 (50)	1 (25)	7 (37)
Sepsis	0 (0)	1 (17)	3 (38)	0 (0)	4 (21)
Prematurity	0 (0)	1 (17)	3 (38)	1 (25)	5 (26)
Intrapartum Asphyxia	0 (0)	1 (17)	2 (25)	0 (0)	3 (16)
Weight >1 kg	0 (0)	3 (50)	1 (13)	1 (25)	5 (26)
APGAR score >3 at 5 mins	0 (0)	3 (50)	1 (13)	2 (50)	6 (32)
Silverman Anderson score >3	1 (100)	4 (67)	5 (63)	2 (50)	12 (63)

#### Monitoring and patient documentation during CPAP use.

Continuous monitoring of heart rate, oxygen saturation and respiratory rate is vital for newborns on CPAP. However this was not available in most NBUs. We found that general clinical monitoring was present in public national referral hospitals and private hospitals (apart from oxygen saturations) but was lacking in other public hospitals. Overall, this meant that continuous monitoring of heart rate was available in 63% of NBUs, continuous monitoring of oxygen saturations was available in 58% of NBUs, and continuous monitoring of respiratory rate was available in 42% of NBUs. In one public hospital it was reported that pulse oximeters were only available if provided by as personal property by individual clinicians as they were not provided by the hospital. Regular assessment of respiratory distress was less common, occurring in only 8/19 NBUs (42%). Most units had a predefined initial gas flow (84%) and all units had a target oxygen saturation range (100%) (Table 7**).**

**Table 7:** Monitoring of CPAP use in newborns, per hospital type.

Process	National Referral Hospital n (%)	Regional Referral Hospital n (%)	County Referral Hospital n (%)	Private Hospital n (%)	Total n (%)
Continuous monitoring of heart rate (HR)	1 (100)	3 (50)	4 (50)	4 (100)	12 (63)
Continuous monitoring of oxygen saturation (SpO2)	1 (100)	3 (50)	4 (50)	3 (75)	11 (58)
Continuous monitoring of respiratory rate (RR)	1 (100)	2 (33)	4 (50)	1 (25)	8 (42)
‍Regular assessment of respiratory distress	1 (100)	2 (33)	4 (50)	1 (25)	8 (42)
Use of predefined initial gas flow	1 (100)	6 (100)	7 (88)	2 (50)	16 (84)
Use of predefined oxygen saturation range	1 (100)	6 (100)	8 (100)	4 (100)	19 (100)

In many of the public hospitals, the documentation of monitored patient observations was limited by the absence of patient observation charts (Table 8). When asked about the recording of observations in the patient notes, 74% of NBUs recorded cardiac and respiratory rate but only 44% recorded oxygen saturation. Only 3 NBUs (16%) recorded respiratory distress in the patient notes. Recording of CPAP settings, such as CPAP pressure, gas flow, gas humidity, and gas temperature, was poor at 26%, 26%, 5% and 11% respectively. The county referral and regional referral hospitals were least likely to record appropriate observations for safe CPAP use in the public health sector, which was consistent with the lack of clinical observation charts in many of these hospitals.

**Table 8:** Documentation of CPAP use in newborns, per hospital type.

Process	National Referral Hospital n (%)	Regional Referral Hospital n (%)	County Referral Hospital n (%)	Private Hospital n (%)	Total n (%)
Recording of heart rate (HR)	1 (100)	4 (67)	5 (63)	4 (100)	14 (74)
Recording of oxygen saturation (Sp02)	1 (100)	1 (17)	2 (25)	4 (100)	8 (42)
Recording of respiratory rate (RR)	1 (100)	4 (67)	5 (63)	4 (100)	14 (74)
Recording of respiratory distress	1 (100)	0 (0)	0 (0)	2 (50)	3 (16)
Recording of CPAP pressure	1 (100)	0 (0)	1 (13)	3 (75)	5 (26)
Recording of gas flow	1 (100)	0 (0)	1 (13)	3 (75)	5 (26)
Recording of gas humidity	0 (0)	0 (0)	0 (0)	1 (25)	1 (5)
Recording of gas temperature	1 (100)	0 (0)	0 (0)	1 (25)	2 (11)

#### Monitoring for complications and weaning guidance.

Once newborns were on CPAP, most NBUs monitored their nasal skin integrity (95%) but less than half of the NBUs monitored their abdominal girth (43%). CPAP weaning guidelines outlining a standardised process for removing a newborn from CPAP only existed in 47% of NBUs (Table 9).

**Table 9:** Monitoring for complications and weaning process with CPAP use, per hospital type.

Process	National Referral Hospital n (%)	Regional Referral Hospital n (%)	County Referral Hospital n (%)	Private Hospital n (%)	Total n (%)
Monitoring of nasal skin integrity	1 (100)	6 (100)	7 (88)	4 (100)	18 (95)
Monitoring of abdominal girth	1 (100)	2 (33)	2 (25)	3 (75)	8 (43)
Standardised CPAP weaning process	1 (100)	2 (33)	2 (25)	4 (100)	9 (47)

#### Adherence to infection control guidance.

The adherence to processes related to infection control was low, especially in public regional or county hospitals. This meant that 53% of NBUs used CPAP-specific breathing circuits for one patient only, 53% of NBUs used nasal interfaces for one patient only and 58% of NBUs removed water condensation from CPAP tubing. These three processes are important in reducing the risk of sepsis related to use of CPAP use (Table 10).

**Table 10:** Adherence to infection control guidance with CPAP use in newborns, per hospital type.

Process	National Referral Hospital n (%)	Regional Referral Hospital n (%)	County Referral Hospital n (%)	Private Hospital n (%)	Total n (%)
CPAP-specific breathing circuits used for one patient only	1 (100)	3 (50)	2 (25)	4 (100)	10 (53)
Nasal interface for one patient only	1 (100)	3 (50)	2 (25)	4 (100)	10 (53)
Water condensate removed from tubing	1 (100)	2 (33)	5 (63)	3 (75)	11 (58)

## Discussion

Although the implementation of CPAP in NBUs in public hospitals in Kenya begun in 2016 [38], we found that inequitable access to CPAP in NBUs across Kenya was still a challenge in 2017–2018. CPAP use was concentrated in certain parts of the country, predominantly in Western Kenya and in the most populous county (Nairobi country). The provision of CPAP in public hospitals was lower than in private hospitals and CPAP services were better developed in the national hospital as compared to the regional and county referral hospitals. Overall, no hospital achieved all the international standards for infrastructure and human resource expected for safe CPAP use in newborn care. We also found that adherence to essential standard of care processes was limited, especially in regional and county referral hospitals.

We found that there were significant gaps in the general infrastructure needed to support CPAP use, including reliable access to electricity and gas supply. In similar low resource settings across the world, such as India, there is good access to oxygen [12] but in Kenya, parents are often expected to cover the cost of oxygen supply, causing further inequalities in the access to CPAP. Most NBUs in Kenya used commercial CPAP systems rather than home-made systems, which is in line with existing research from other African countries, such as Malawi, that are in the process of scaling-up this intervention in district hospitals [39]. This presents opportunities for standardisation and benchmarking of CPAP implementation that can drive further improvements in the quality of neonatal care within the country [40]. We also found that many NBUs in Kenya were faced with a lack of CPAP-specific infrastructure, often because of poor supply chains for CPAP equipment and consumables. The absence of consumables, such as breathing circuits and nasal prongs meant that the NBUs in Kenya had to reuse single-use consumables for CPAP, potentially increasing the transmission of neonatal infections between infants.

Another key issue highlighted in our results was the lack of CPAP trained staff in NBUs in Kenya. We found that there was often no CPAP trained nurse or doctor available on a shift. Healthcare workers in Kenya frequently rotate between departments, including rotating from newborn care to adult care [29, 41]. These rotations result in the loss of designated “CPAP champions”, as well as a wider loss of confidence in using CPAP by the remaining members of the healthcare team. The original training of staff to use CPAP in Kenya was supported by international partners when CPAP devices were introduced [38], but we found that this training was not continued after these programmes ceased. We also found that there was a low nurse to infant ratio in most NBUs, which meant that appropriate monitoring of infants on CPAP was difficult to maintain. This has been reported in other contexts in Africa [24], including Malawi where a CPAP trial had to be stopped prematurely due to safety concerns related to health system limitations [23].

There was significant disruption of newborn care services due to recurrent healthcare worker strikes during this evaluation. In some hospitals, this resulted in the discontinuation of CPAP use as skills and confidence diminished because of loss of staff and disruption of procurement processes. A time series descriptive analysis of attendance for maternal and child health services in Kilifi County by Mohiddin et al found that the drop in activity levels in the public sector were only partially compensated by an increase in non-public sector activities [42]. However, although there were some post-strike catch-up activities, these were often too late to mitigate the disastrous impact that the strike had on maternal and child health services and outcomes [42].

We found that many essential medicines typically used along with CPAP, such as caffeine citrate for the management of severe respiratory distress syndrome among preterm infants, were not available in most NBUs in Kenya, therefore likely limiting CPAP's impact on improving newborn survival [43]. Interestingly hospital level support services, such as access to laboratory and radiological investigations and provision of basic emergency equipment was good. However, there was a lack of more specialist equipment for the identification and treatment of the common sequela of CPAP use, such as pneumothorax. The availability of ventilators for infants that deteriorated on CPAP was poor, meaning that there was limited ability to escalate treatment if needed when CPAP failed.

We found that there was significant variation in the processes for CPAP use. There were national newborn care protocols in Kenya at the time of our study [27], however, these contained limited guidance about monitoring infants on CPAP and when to cease CPAP. As a result, there was a wide variation in the criteria used by different NBUs when initiating and weaning infants on/off CPAP. There was also poor monitoring of infants and documentation of their observations whilst on CPAP, as well as limited adherence to infection control guidance when using CPAP equipment. The current neonatal care protocols in Kenya provide more comprehensive guidance for CPAP use and recommend it as the standard of care for preterm infants in level 2 newborn units as per WHO Every Newborn Action Plan guidance [13, 28].

In summary, we found that there is scope for further scale up of the use of CPAP in Kenya, however, this needs to occur alongside a full package of care to support the safety of this intervention. The fact that the original introduction of CPAP in Kenya was donor funded, with no national standardisation of services, may have explained some of the infrastructure and process discrepancies identified in our data. There is growing evidence about the potential benefits of CPAP in improving RDS management in resource limited settings [44, 45]. However, the use of CPAP alone as a ‘magic bullet’ without sufficient infrastructure or processes is unlikely to reduce neonatal mortality and may lead to increased morbidity in this patient group [29].

Based on our results, there is an urgent need to ensure that essential newborn care practices are optimised as CPAP scale-up continues across Kenya, otherwise neonatal survival is unlikely to improve. The safe use of CPAP is dependent on many wider support services and resources as outlined in our results. It is vital to repeat our survey to determine any recent developments in the infrastructure and processes that currently exist in Kenya and link this to newborn outcomes. A new survey would guide national procurement of equipment and essential medication related to CPAP in order to reduce these previously observed inequalities in CPAP use across the country. The implementation of a relevant package of care should involve the dissemination of the Kenya Neonatal Care Protocol for CPAP use, as well as a programme of regular staff training to help maintain the skills and confidence of rotating healthcare workers using CPAP. It is important to improve the retention of skilled staff through greater recognition of the neonatal nurses via appropriate remuneration and continuing professional development, for example by expanding the existing “training-of-trainer” model used for newborn care in the country [38]. Overall, ongoing public and private health sector partnership of international alliances, notably NEST360 [40], should be encouraged to continue to help promote the safe, effective and sustainable scale up of the use of CPAP in NBUs across Kenya. This activity needs to be targeted specficially on improving the capacity of regional and county hospitals in Kenya given their poor performance in our results.

## Strengths and limitations

Our main strength was that we were able to collect comprehensive data about the use of CPAP in newborn units across Kenya. This data collection occurred 4 years after a comprehensive private partnership scale up programme of CPAP (led by the Centre for Public Health and Development) across public health hospitals in Kenya [38]. In 2019, after our survey, the NEST360 project was established in Kenya and has been working with 13 county referral hospitals in the country [40]. This ambitious private-public partnership seeks to support the Ministry of Health in Kenya to implement the WHO Every Newborn Action Plan (ENAP) [46]. A key ENAP strategy is to ensure that CPAP is routinely used in at least 80% of County referral hospitals by 2025 [46].

Our main limitation was that these data were collected seven years ago, before the COVID-19 pandemic, and does not necessarily reflect the current situation of all newborn units in Kenya. There is limited published data on CPAP use in newborn care in Kenya [45]. Indeed, although the Ministry of Health guidelines state that CPAP use should be standard of care for preterm infants with RDS, the coverage in Kenya remains inequitable despite input by NEST360 and other private/public initiatives (e.g., USAID) [47]. The majority of NEST360 hospital facilities are located in southern Kenya meaning there continues to be limited CPAP use in northern Kenya [40]. A recent quality improvement initiative in Nakuru County, Kenya published in 2020 was unable to demonstrate any change in mortality when implementing a bubble CPAP treatment program for sick newborns [48]. This paper concluded that CPAP use was safe and feasible in low resource settings; however, identified various gaps in the CPAP care process, including the fact that training interventions did not adequately teach staff to provide effective CPAP treatment [48]. Another recent publication based in Kenya in 2021 highlighted similar barriers to CPAP including inadequate number of CPAP machines, inadequate training and mentorship, inadequate CPAP consumables, staff shortages, long servicing and cleaning turnaround times, infracture challenges and insufficient utilities [49]. Interestingly future research is currently planned in Kenya to determine the effect on the quality of patient care of adding extra nursing staff to newborn units in order to better describe the relationship between staffing and quality of care [50]. The launch of Phase 2 of NEST360s work in January 2024 [40] means that provision of more CPAP machines across newborn units in Kenya is likely to be implemented soon. Given that the scaling up of high-quality neonatal care has been estimated to cost approximately US$93 000 per level 2 hospital using planning and costing tools in Tanzania [51], it is important that future international funding is focused on interventions that are both feasible and safe in low resource settings. Therefore we feel it was important to publish our data before this next phase of NEST360 as our data provides a valuable baseline data about the pre-NEST360 provision of CPAP in newborn care units in Kenya. Our data is relevant to other low and middle income countries that are starting to acquire CPAP on the African continent [52, 53].

Our other main limitation was that we collected our survey data during a period of healthcare worker strikes that disrupted newborn care services across Kenya. Our questionnaire was designed to describe CPAP use in NBUs in Kenya despite the strikes, so we were still able to accurately collect our survey data. However, these strikes did mean that we were unable to reliably link our data about CPAP use to neonatal outcomes as planned, because many NBUs did not use CPAP routinely during this period.

Also, regarding infection control measures the apparent low adherence across NBU’s may be due to the fact that the tubings and the prongs available were for repeated use versus single use that would be standard practice in high income countries [54]. However, we were not able to get reliable information on whether the circuits were single use or for repeated use, as most of these had been provided through donor-led projects and once these had ended, the local teams did not appear to have clear details about the procurement process for the consumables or if there was a process to decontaminate the reusable ones.

Finally, we were unable to complete thorough subgroup analysis between the different hospital types due to the small number of national referral and private hospitals in our data.

## Conclusion

Our data highlights multiple issues related to the limited infrastructure and processes required for safe and effective use of CPAP in in the public hospitals in Kenya. In addition, the provision of CPAP in NBUs in Kenya was limited to less than a third of counties rendering this service inequitable.. Establishing national standards and norms to address the disparities in resources and process of CPAP use between hospitals in Kenya is urgently needed as the country works towards ending preventable newborn deaths.

## Supporting information

S1 Fig Availability of CPAP newborn care in public hospitals in Kenya. (DOCX)

S1 Data Inclusivity questionnaire. (DOCX)
