# Supplementary material for: A survey on the use of continuous positive airway pressure in newborn care in Kenya in 2017–2018
Source: PLoS One. 2025 Apr 30;20(4):e0322310. doi: 10.1371/journal.pone.0322310 (PMC12043177; doi:10.1371/journal.pone.0322310)
Supplement: S1 Fig — (DOCX) [file pone.0322310.s001.docx]

**Fig S1: Availability of CPAP newborn care in public hospitals in Kenya**

**Regions of Kenya: 8**

**Public Newborn Units: ~47**

**Public Newborn Baby Units providing**

**CPAP: 18 (~38%)**

**Used to pilot the tools: 1**

**Declined participation: 2**

**Public Newborn Baby Units providing CPAP included in the survey: 15 (82%)**

**Level 6 (National referral): 1**

**Level 5 (Regional referral): 6**

**Level 4 (County referral): 8**
